# Left-Sided Gastroschisis with Meckel’s Diverticulum: A Rare Presentation

**DOI:** 10.21699/jns.v6i3.502

**Published:** 2017-08-10

**Authors:** Aditya Pratap Singh, Vinay Mathur, Ramesh Tanger, Arun Kumar Gupta

**Affiliations:** Department of Surgery (Pediatric Surgery), SMS Medical College Jaipur, Rajsthan, India

**Dear Sir**

A 1-day-old female baby, the product of spontaneous vaginal delivery weighing 2.0 kg and delivered to 22 years old primipara, presented to the emergency room with evisceration of intestine from abdomen. Prenatally she was diagnosed with gastroschisis. On examination, defect was about 2 cm × 0.5 cm in size on the left side of umbilicus with evisceration of intestine. The eviscerated bowel was oedematous and dusky in colour. Patient was stabilised by putting him in warmer, oxygen supply and adequate intravenous fluids. After stabilisation patient was shifted to the operation theatre. Midline upper abdominal incision was extended to the defect laterally on left side. Intraoperative findings showed presence of Meckel’s diverticulum and there was no distinction between large and small bowel. There was no situs inversus. Mechanical stretching of anterior abdominal wall was done. The eviscerated bowel was reduced into the abdominal cavity. Skin closure of the defect was performed. Postoperative recovery was uneventful.

**Figure:**
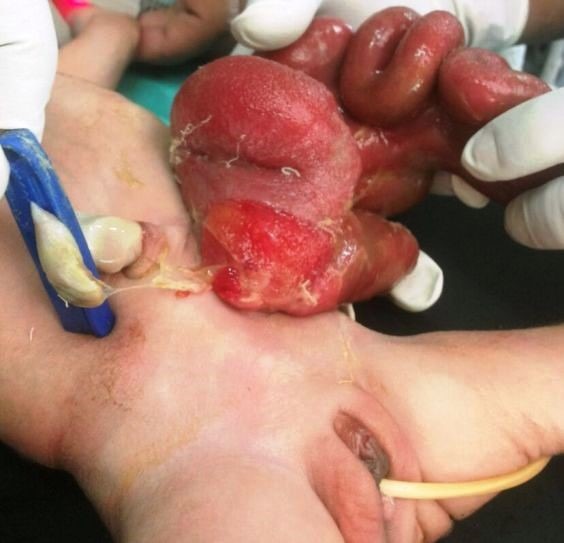
Figure 1: Showing defect lateral to the umbilicus.

Gastroschisis is a congenital anomaly characterised by a defect in the anterior abdominal wall through which the intestinal contents freely protrude. Defect is located mostly to right of umbilicus. Few cases of left-sided gastroschisis have been reported in literature. Associated anomalies are rare but intestinal atresia is present in up to 15% of cases. Review of literature suggests that the incidence of extra intestinal congenital anomalies is significantly higher in left-sided gastroschisis as compared to right sided gastroschisis [1-4]. Reported extra intestinal anomalies include choledochal cyst, cleft lip, cleft palate, pulmonary hypoplasia, atrial septal defect and patent ductus arteriosus, etc. [1,3]. However, in our case no extra intestinal anomalies were detected. We report a case of left-sided gastroschisis associated with Meckel’s diverticulum. The defect was not only to the left side but also inferior to the umbilicus which makes it even more rare 

## Footnotes

**Source of Support:** None

**Conflict of Interest:** None
